# Taxonomic authentication and phytochemical characterization of an endangered medicinal species, *Cordia macleodii* (Boraginaceae), for pharmaceutical advancement and conservation in Central India

**DOI:** 10.3389/fpls.2026.1945739

**Published:** 2026-09-23

**Authors:** Kaushal Tripathi, Shakti Nath Tripathi, Nikhil Verma, Fatima Shirin, Arpita Tripathi, Claudia Balint, Aurel Maxim, Petru Burduhos, Ioan Bănăţean-Dunea, I. Valentin Petrescu-Mag, Praveen Pandey

**Affiliations:** 1 Genetics and Tree Improvement Division, ICFRE-Tropical Forest Research Institute, Jabalpur, Madhya Pradesh, India; 2 Department of Botany, Nehru Gram Bharati Deemed to Be University, Prayagraj, India; 3 Faculty of Education, Teerthanker Mahaveer University, Moradabad, India; 4 Department of Environmental Engineering and Protection, Faculty of Agriculture, University of Agricultural Sciences and Veterinary Medicine, Cluj-Napoca, Romania; 5 EBBSA International, Niš, Serbia; 6 Biology and Plant Protection Department, Faculty of Agriculture, University of Life Sciences King Mihai I” from Timişoara, Timişoara, Romania; 7 Doctoral School of Engineering Science, University of Oradea, Oradea, Romania; 8 Division of Plant Breeding and Genetic Resource Conservation, CSIR-Central Institute of Medicinal and Aromatic Plants, Lucknow, India

**Keywords:** Dahiman, deciduous tree, exalbuminous seed, phenology, phytochemical profiling, secondary metabolites

## Abstract

**Introduction:**

*Cordia macleodii* (Griff.) Hook. f. & Thomson (Boraginaceae), locally known as Dahiman, is an endangered medicinal tree of Central India. Accurate taxonomic authentication and systematic phytochemical characterization are important for its conservation and for evaluating its potential medicinal applications. This study aimed to integrate taxonomic characterization with qualitative and quantitative phytochemical assessment of *C. macleodii* leaves and bark.

**Methods:**

Field surveys were conducted between 2021 and 2024 across 24 forest divisions in Madhya Pradesh, Chhattisgarh, and Maharashtra, resulting in the identification and documentation of 262 individuals. Morphological characters were examined and compared with herbarium specimens to establish diagnostic traits. Leaf and bark samples were extracted using 70% ethanol, 70% methanol, and distilled water. Qualitative screening was performed for major phytochemical groups, while total phenolic content (TPC) and total flavonoid content (TFC) were quantified using catechol and quercetin as standards, respectively.

**Results:**

Morphological examination confirmed distinctive diagnostic characteristics, including light brown papery bark, cordate-based ovate leaves with a densely tomentose abaxial surface, pentamerous or hexamerous flowers, drupaceous fruits with a single exalbuminous seed, and dioecious floral structures. Flowering commenced in the first week of March, with anthesis observed between 07:30 and 10:30 and butterfly-mediated pollination. Phytochemical screening revealed alkaloids, tannins, coumarins, phenols, flavonoids, saponins, phlobatannins, glycosides, sterols, terpenoids, quinones, and oxalates, with substantial variation according to plant tissue and extraction solvent. The highest TPC was recorded in the ethanolic bark extract (11.72 mg CE/g), whereas the highest TFC was detected in the methanolic bark extract (12.19 mg QE/g) .

**Discussion:**

The integrated taxonomic and phytochemical assessment provides reliable morphological markers for field identification and demonstrates substantial variation in the secondary metabolite profile of *C. macleodii* according to tissue and solvent. The findings support the ethnomedicinal relevance of the species and provide a scientific basis for its conservation, sustainable propagation, and further pharmacological investigation.

## 1 Introduction

Angiosperms, which account for nearly eighty percent of all documented plant species globally, are the most ubiquitous and evolutionarily successful group of living seed plants. They encompass a broad variety of living forms, from little aquatic plants and delicate herbs to substantial shrubs, climbers, and enormous forest trees, and they display exceptional evolutionary adaptability (Tripathi et al., 2023). Angiosperms are essential to preserving ecological balance and supporting human life because of their great diversity and flexibility (Evert et al., 2013; Petrescu-Mag, 2026). They provide vital ecosystem services such oxygen production, carbon sequestration, soil consolidation, and nutrient cycling in addition to serving as the main sources of food, medicine, fiber, and fuel (Sulis et al., 2025; Singh et al., 2006; Pandey et al., 2026a).

Planning for conservation, quantifying biodiversity, and employing plant resources sustainably all require accurate taxonomic identification of angiosperm species (Simpson, 2019). Traditional plant taxonomy, which is primarily based on the morphological properties of vegetative and reproductive organs, continues to form the backbone of plant systematics and is still crucial for field identification and floristic studies, despite notable advancements in molecular biology and phylogenetics (Judd et al., 2016). The importance of phytochemical research of angiosperms is growing alongside taxonomic studies due to the increasing demand for plant-based medications and natural goods (Harborne, 1998; Pandey and Tripathi, 2014; Kumari et al., 2025; Wang et al., 2024). Alkaloids, flavonoids, tannins, phenolics, terpenoids, glycosides, and saponins are merely a few of the various secondary metabolites that plants synthesize (Simion et al., 2025; Lutfi et al., 2023). Most of these compounds have strong biological activities, including antioxidant, antimicrobial, anti-inflammatory, and anticancer properties (Kokate et al., 2014; Verpoorte et al., 2005). In conjunction with supporting conventional medical claims, phytochemical screening offers a scientific foundation for drug development and uniformity (Fabricant and Farnsworth, 2001; Jangde et al., 2026). Therefore, for accurate species authentication and methodical investigation of therapeutic potential, an integrated strategy combining taxonomic identification with phytochemical profiling is needed.

*Cordia* L. is an important genus of the family Boraginaceae (subfamily Cordioideae), comprising over 300 species distributed predominantly in tropical and subtropical regions across Africa, South Asia, and tropical America, exhibiting a pantropical distribution (Mabberley, 2008). In India, the genus is represented by sixteen species (Santapau and Henry, 1973), among which *Cordia macleodii* (Griff.) Hook. f. & Thomson is a medium-sized deciduous tree of considerable ethnomedicinal importance. Early floristic records report its occurrence in Central India and the Chota Nagpur Plateau (Griffith, 1843; Clarke, 1883; Prain, 1903). The species *C. macleodii*, locally known as Dahiman, is more familiar to indigenous communities than to researchers, and precise identification at the field level often remains problematic. About 250 individuals of *C. macleodii* known by a variety of colloquial names were found in Madhya Pradesh (MP), Chhattisgarh (CG), and Maharashtra (MH). According to recent research, *C. macleodii*’s close resemblance to other *Cordia* species and its increasing ethno-medical use make accurate taxonomic identification necessary to prevent adulteration and misidentification (Sikarwar and Chopade, 2024). Because of its physical similarities to other trees for example, its leaves like those of *Gmelina arborea* and its bark resembles those of *Butea monosperma* identifying this species in its natural habitat is particularly difficult. The necessity of precise taxonomic authentication is further highlighted by this morphological overlap.

*C. macleodii* is a rich source of biologically active secondary metabolites, such as flavonoids, phenolics, alkaloids, terpenoids, glycosides, and sterols, which together contribute to its varied pharmacological properties, according to recent phytochemical studies (Adile and Seth, 2024; Chaubey et al., 2015). Concurrently, *C. macleodii*’s medicinal potential has been highlighted by growing pharmacological research. Several significant bioactive compounds have been found in its leaves and bark, according to preliminary phytochemical research (Qureshi et al., 2009; Nariya et al., 2010, 2012; Chandrakar et al., 2019). Its chemical makeup and high *in vitro* antioxidant capability have been thoroughly revealed by sophisticated chromatographic and spectroscopic methods including GC-HR-MS and FTIR, creating a solid scientific basis for drug discovery (Adile and Seth, 2024). Later studies have shown hepato-protective, antioxidant, antimicrobial, anti-inflammatory, wound-healing, antivenom, and toxicity-modulating properties (Chandrakar and Dixit, 2017; Soni and Bodakhe, 2014; Bhide et al., 2011a, 2011b; Pandya et al., 2014).

In view of the above, the present study aims to provide a comprehensive taxonomic identification of *C. macleodii* along with qualitative phytochemical characterization of leaf and bark extracts using different solvent systems. Such an integrated approach is expected to contribute to accurate species authentication, support conservation efforts, and facilitate the exploration of this medicinally important species for potential applications in herbal medicine and pharmaceutical development.

## 2 Materials and methods

### 2.1 Survey and geo-referencing

An extensive field surveys were carried out between 2021 and 2024, during which total of 262 Dahiman trees were identified across 24 forest divisions in the central Indian states of Madhya Pradesh (21.3–24.8°N, 74.0–82.8°E), Chhattisgarh (17.8–24.1°N, 80.2–84.4°E), and Maharashtra (16.0–22.1°N, 72.6–80.9°E) (**Table 1**). Field studies were conducted in multiple seasons. For each individual, observations were made on plant habitat, growth form, approximate height, stem characteristics, leaf morphology, flowering and fruiting status, regeneration, and associated vegetation of the species. The collected field data were systematically compiled and analyzed to determine the spatial distribution of the species. Based on the recorded GPS coordinates, a detailed distribution map was subsequently prepared using ArcGIS software. During each survey, the latitude and longitude of every collection site were recorded to ensure accurate documentation of the sampling locations. Photographs documenting the plants at different stages were taken.

**Table 1:** Tree identified and marked in different forest divisions of Chhattisgarh, Madhya Pradesh and Maharashtra.

State	No. of trees	Locations	Year
CG	151	Marvahi, Bilaspur, Sarguja, Ambikapur, Katghora, Raigarh, Manendragarh, Korea, Dhamatari, Kanker, Jagadalpur	2021-2024
MP	69	Mandla, Jabalpur, Katni, Damoh, Rewa, Satna, Sahdol, Sagar, Anuppur, Singarauli	2023-2024
MH	42	Gondia, Pune, Bhimashankar	2023-2024

### 2.2 Collection of plants materials and examination of type specimens

For the analysis of phytochemicals present in Dahiman, plant materials (bark and leaves) were collected from forest division of Chhattisgarh in the month of May 2022. Information associated with herbarium specimens of *C. macleodii* (Dahiman) deposited at the Botanical Survey of India, Central Regional Centre, was examined and compiled (**Table 2**). The preliminary information obtained from these sources was used for the rapid identification and localization of Dahiman populations during field surveys conducted in the forest divisions of Chhattisgarh (CG), Madhya Pradesh (MP), and Maharashtra (MH).

**Table 2:** Herbarium specimens of *Cordia macleodii* collected from central India.

Herbarium	Collector	Collection date	Collection No.	Barcodes No. (BSA00000--)	Accession number	Location
BSA	Dr. A K Verma & B Lakshaman	30.03.20222	80327	75673	106953	Kuno National Park
BSA	Dr. A K Verma & B Lakshaman	30.03.20222	80327	75671	106955	Kuno National Park
BSA	G. Panigrahi	4.01.1971	13930	85896	22911	Duramalganj, Mirzapur
BSA	G. Panigrahi	13.04.1965	8502	–	10565	Katghora
BSA	Bhaskar Datta	18.02.1982	33716	85894	86662	Chhatarpur
BSA	Bhaskar Datta	18.04.1982	33716	85893	86731	Chhatarpur
BSA	Bhaskar Datta	1963	33579	85903	86635	Chhatarpur
BSA	Bhaskar Datta	18.06.1982	33716	85900	86803	Chhatarpur
BSA	S. M Mishra	02.08.1964	3831	85898	7988	Bilaspur
BSA	K. P Singh, P K Sahu, A N Shukla	18.10.2007	68843	85895	77789	AABR, Chhattisgarh
BSA	K. P Singh, P K Sahu, A N Shukla	18.10.2007	68843	85904	77788	AABR, Chhattisgarh
BSA	R K Baneyie	17.03.1978	28210	85911	38743	Seoni, M P
BSA	R K Baneyie	17.03.1978	28210	85910	38742	Seoni, M P
BSA	Rampal	21.02.1985	37002	85909	74937	Panna, MP
BSA	Rampal	21.02.1985	37002	85907	61061	Panna, MP
BSA	Rampal	21.02.1985	37002	85908	61060	Panna, MP
BSA	Rampal	24.03.1983	34417	85905	53404	Panna, MP
BSA	Rampal	24.03.1983	34417	85906	53403	Panna, MP
BSA	A P Tiwari	23.04.2011	69144	85901	89288	Ranipur WS, UP

### 2.3 Taxonomic identification

Extensive field excursions were used to gather specific information about habit, habitat, leaves, stem, color, shape, fruit and blossom size, bark type, etc. Several floras and pertinent taxonomic literature were used to identify the species (Hooker, 1858; Duthie, 1903-1922; Brandis, 1906; Singh, 1997).

### 2.4 Preparation of extracts

To get a consistent powder, the dried plant materials were first crushed into a fine powder using a mechanical grinder. Using 70% ethanol, 70% methanol, and distilled water as extraction solvents, ten grams (10 g) of the powdered plant material were weighed separately. To ensure comparability between the various solvent extracts, each extraction was performed independently under the same conditions. To enable the effective diffusion of bioactive components into the appropriate solvents, the extraction solutions were kept at room temperature and continuously shaken on an orbital shaker. Each mixture was meticulously filtered through Whatman No. 1 filter paper to eliminate insoluble plant debris and produce clean filtrates when the extraction procedure was finished. In order to eliminate the solvents and minimize the potential thermal destruction of heat-sensitive phyto-constituents, the filtrates were further concentrated under decreased pressure at 45 °C using a rotary evaporator. The corresponding crude extracts were obtained by further drying the concentrated extracts that were produced. Each dried crude extract was precisely weighed and reconstituted in 0.1 N NaCl to achieve a final stock solution concentration of 1 mg mL^−^¹ for the phytochemical and biological analyses that followed. The produced stock solutions were employed for additional experimental analyses after being suitably labeled based on the plant material and extraction solvent. The process stated by Ahmed et al. (2020) was followed for the extraction and preparation of the stock solutions, with the necessary adjustments made as needed.

### 2.5 Qualitative assessment of phytochemicals

Following standard procedures (**Table 3**) with some modifications, the freshly prepared crude ethanol (EE), methanol (ME), and distilled water (DE) extracts were qualitatively examined for the presence of phytochemicals (**Table 4**).

**Table 3:** Procedures and indicators for the detection of phytochemicals in plant parts.

S. No.	Phytochemical	Procedure	Positive indicator	Reference
1	Alkaloids (Wagner’s reagent test)	1 mL of each extract was transferred into test tubes, and 4–6 drops of Wagner’s reagent (prepared with 1.27 g iodine and 2 g potassium iodide dissolved in 100 mL distilled water) were added.	Reddish-brown precipitate	Rizk, 1982
2	Coumarins	1 mL of the extract was mixed with 1.5 mL of 10% NaOH solution.	Yellow coloration	Ugochukwu et al., 2013
3	Glycosides (Keller–Killiani test)	1 mL of each extract was mixed with 1 mL of glacial acetic acid, followed by the addition of 5–6 drops of 1% ferric chloride solution.	Brown-colored ring at the upper layer of the solution	Ahamed et al., 2017
4	Tannins (Braymer’s test)	1 mL of each extract was mixed with 1 mL of distilled water, followed by the addition of 4–5 drops of 1% ferric chloride solution.	Blue or greenish-black coloration	Ahamed et al., 2017
5	Phenols (Ferric chloride test)	1 mL of each extract was mixed with 1 mL of ethanol, followed by the addition of 5–8 drops of 1% ferric chloride solution.	Green, blue, or purple coloration	Soloway and Wilen, 1952
6	Flavonoids (Alkaline reagent test)	2 mL of each extract was mixed with 2–5 drops of 20% NaOH solution. The developed yellow color disappeared after the addition of 4–5 drops of dilute HCl.	Intense yellow coloration	Ugochukwu et al., 2013
7	Saponins (Foam test)	1 mL of each extract was mixed with 2 mL of distilled water and vigorously shaken for 5 min.	Formation of persistent thick foam	Kumar et al., 2009
8	Phlobatannins (Precipitate test)	2 mL of each extract was boiled with 1 mL of 1% aqueous hydrochloric acid.	Red precipitate	Ugochukwu et al., 2013
9	Sterols (Liebermann–Burchard test)	1 mL of each extract was mixed with chloroform, acetic anhydride, and concentrated H_2_SO_4_.	Dark pink or red coloration	Ugochukwu et al., 2013
10	Terpenoids (Salkowski’s test)	2 mL of each extract was mixed with 1 mL of chloroform, followed by the addition of a few drops of concentrated H_2_SO_4_.	Reddish-brown precipitate	Ugochukwu et al., 2013
11	Quinones	A small amount of each extract was treated with concentrated HCl.	Yellow precipitate	Ugochukwu et al., 2013
12	Oxalates	3 mL of each extract was mixed with a few drops of glacial acetic acid.	Greenish-black coloration	Ugochukwu et al., 2013.

**Table 4:** Phytochemical screening using different *C. macleodii* extracts.

Phytochemical	Ethanolic extract (EE)		Methanolic extract (ME)		Distilled water (DW)	
	Leaf	Bark	Leaf	Bark	Leaf	Bark
Alkaloids	+	+	+	+	+	+
Glycosides	_	+	+	+	_	+
Coumarins	+	+	+	+	+	+
Tannins	+	+	+	+	+	+
Phenols	+	+	+	+	_	+
Flavonoids	+	+	_	+	+	+
Phlobatannins	_	+	+	+	+	+
Saponins	+	+	_	+	_	+
Sterol	_	_	_	_	+	+
Terpenoids	_	_	_	_	+	+
Quinones	_	+	_	+	+	+
Oxalate	+	_	+	_	_	_

### 2.6 Quantification of total phenolic content

The total phenolic content present in the ethanol, methanol, and distilled water extracts of Dahiman (leaves and bark) was estimated according to the Folin–Ciocalteu method described by Miliauskas et al. (2004). Briefly, 1 mL of the extract was mixed with 0.5 mL of Folin–Ciocalteu reagent. After 6–8 minutes, 1.5 mL of 20% sodium carbonate (Na_2_CO_3_) solution and 10 mL of distilled water were added. The mixture was then incubated at 25 °C for 1 hour. After incubation, a blue color developed, and the absorbance was measured at 750 nm using a UV–visible spectrophotometer. The total phenolic content was determined using catechol as a standard and expressed as milligrams of catechol equivalent per gram of dry sample (mg CE/g).

### 2.7 Quantification of total flavonoid content

The total flavonoid content (TFC) in the ethanol, methanol, and distilled water extracts of Dahiman was estimated according to the method described by Shohael et al. (2006). Briefly, 0.5 mL of the extract was mixed with 1.5 mL of ethanol. After 5 minutes, 0.15 mL of 10% AlCl_3_ solution was added, and the mixture was allowed to stand for an additional 5–6 minutes. Subsequently, 0.1 mL of potassium acetate was added. The final volume of the mixture was adjusted to 2.8 mL with distilled water and mixed thoroughly. The absorbance was measured immediately at 415 nm using a spectrophotometer. The concentration of total flavonoids was determined using quercetin as a standard and expressed as milligrams of quercetin equivalent per gram of extract (mg QE/g).

## 3 Results

As compared to the herbarium specimens kept at the Botanical Survey of India, Central region, specimens of *C. macleodii* collected during extensive surveys across different forest divisions in Central India between 2021 and 2024 (**Figure 1**) showed no morphological variations. **Table 3** provides details as well as references to the herbarium specimens that were looked at.

**Figure 1:**
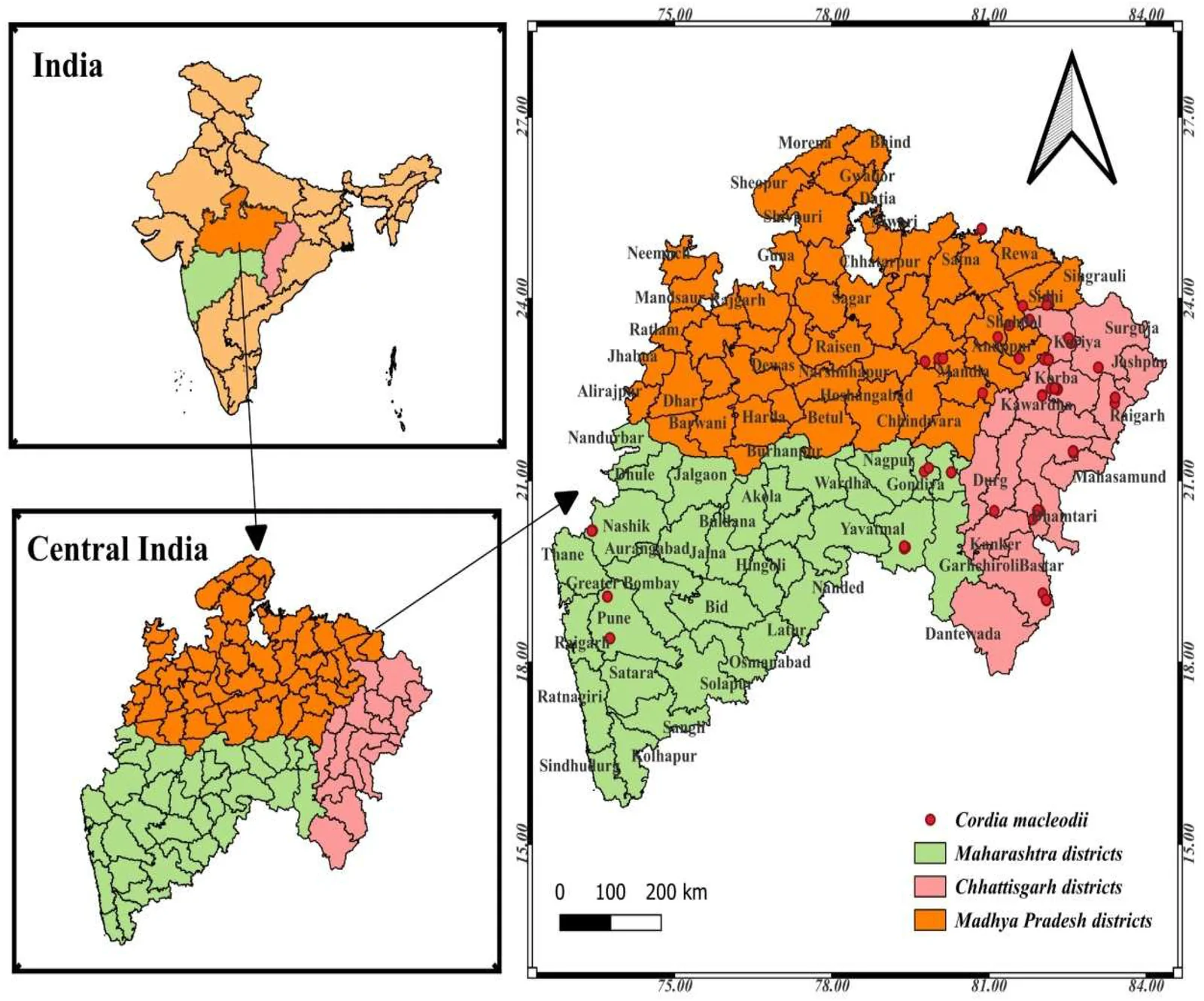
*C. macleodii* individuals identified and marked in different forest divisions of CH, MP and MH during 2021-2024.

### 3.1 Taxonomic description of *C. macleodii*

Dahiman is a medium-sized deciduous tree, reaching a maximum height of 12–14 meters (**Figures 2A, B**). Stem: cylindrical, woody, branched, light brown bark (10–12 mm) with papery chips (**Figures 2C, D**). Leaves: simple, alternate, petiolate (4-6.5 cm), light to dark green, 7–16 cm × 5–12 cm, obtuse-ovate. Abaxial surface of leaf a light green color, densely tomentose with whitish hairs; subentire, slightly undulate, sub-serrate margin in mature leaves; serrate in sapling stage (**Figures 2E–G**). Base: often cordate, 3–5 nerved from base with muticostate divergent, reticulate venation. Leaf fall during the reproductive phase (**Figure 2B**). Inflorescence: racemose, compound corymb type, flowers paniculiform, diameter 5.5–10.2 cm (**Figure 1H**). Polygamous, yellowish-white flowers (**Figures 2I, J, O**); calyx: 5 sepals, gamosepalous, green, tomentose, cupuliform; shorter than the corolla (8–10 mm in flower, 8–12 mm in fruit), penta-hexaribbed, suboblate, persistent (**Figure 2K**); corolla: 5 sepals (**Figures 2M, N**), sometimes 6 sepals (**Figure 2P**); gamopetalous, outer wavy shaped, 8–14 mm diameter, subrotate; corolla tube ligulate-obtuse, 9–10 mm, yellowish-white. Single inflorescence may be contained pentamerous and hexamerous flowers (**Figures 2U, V**). 5 stamens in pentamerous and 6 in hexamerous, including one staminode: free, epipetalous, alternipetalous (alternate with petals) (**Figure 2L**), adnate, and basifixed; filaments 11–12 mm (**Figure 2Q**). Anthers exserted, sagittate, basifixed, diathecous, with rounded, extrose pollen (**Figures 2R, S, T**). Gynoecium: superior ovary, stigma: bifurcated, style: terminal, simple, 1 cm (**Figure 2F**); commonly observed residues in immature fruits (**Figures 2W–Y**); the style was to be absent in male flowers. Ovary: superior, 0.7- 0.8 mm, with erect bifurcated style; four-celled; rudimentary in male flowers. Fruit: drupe, subglobose, yellowish, 1.2–1.9 cm long, somewhat hairy, and sits atop a persistent calyx; ovoid, acute with apical hook (**Figures 2Z, AA**). Seed single, 7–9 mm long, 5–6 mm broad at middle, exalbuminous (**Figure 2BB**). It was also found that the *C. macleodii* tree is distinctly dioecious in nature.

**Figure 2:**
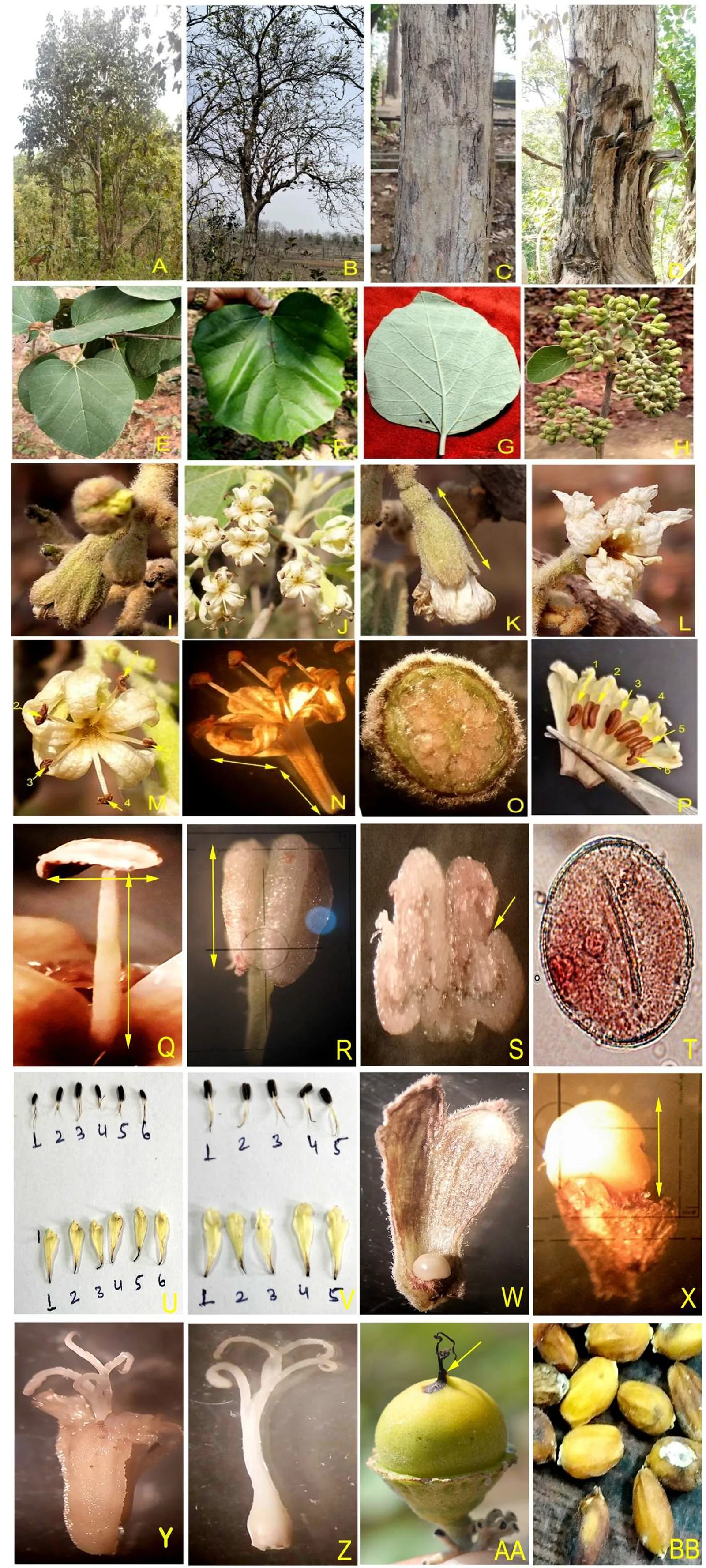
*C. macleodii*: **(A)** Tree in vegetative phase; **(B)** Reproductive phase; **(C)** Stem; **(D)** Wounded bark; **(E)** Normal Leaf (abaxial surface); **(F)** Large leaf; **(G)** Adaxial surface; **(H)** Inflorescence; **(I)** Floral bud; **(J)** Flower; **(K)** Sepal with petal; **(L)** Petals; **(M)** Pentamerous and epipetalous condition; **(N)** Tubular and non-tubular petal condition; **(O)** T. S. of floral bud; **(P)** Hexamerous flower; **(Q)** Androecium; **(R)** Dithecous, bilobed anther with filament; **(S)** T.S. of Anther; **(T)** Pollen grain; **(U, V)**. Pentamere and hexamere; **(W, X)**. Superior ovary without stigma (Male flower); **(Y)** Female flower; **(Z)** Gynoecium (stigma, style, and ovary); **(AA)** Fruits with persistent stigma; **(BB)** Seeds.

### 3.2 Phenology

*C. macleodii* exhibits distinct vegetative and reproductive phases. During the reproductive phase, flowering branches shed their leaves from the tree. An alternating fruiting pattern was observed. Flowering commenced in the first week of March, while some trees simultaneously began to produce fruits. According to reports, anthesis occurs between 7:30 and 10:30 AM, and pollination is carried out by butterflies (**Figure 3**).

**Figure 3:**
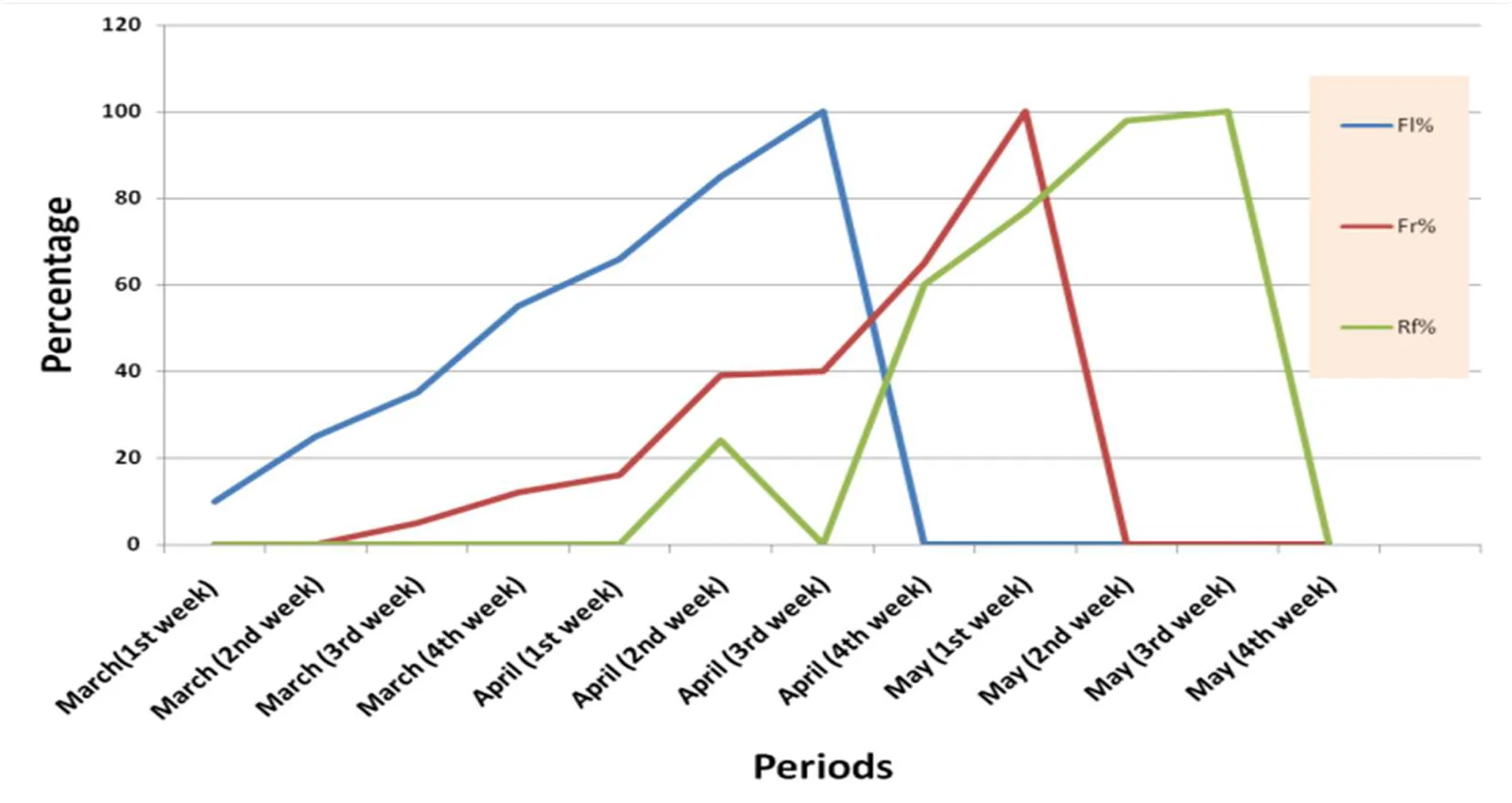
Time scale of flowering, fruiting and fruit ripening condition in forest area.

### 3.3 Screening of phytochemicals

The leaves and bark of *C. macleodii* were subjected to preliminary phytochemical screening for twelve different chemical constituents using various solvents (ethanol extract, EE; methanol extract, ME; and distilled water extract, DW). The results of the preliminary phytochemical screening are summarized in **Table 5**, indicating the presence of several phytochemical constituents in the analyzed extracts. In the ethanolic extracts, sterols and terpenoids were not detected in either the leaf or bark extracts, whereas glycosides, phlobatannins, and quinones were detected in the bark extract but not in the leaf extract. Similarly, sterols and terpenoids were absent in both leaf and bark methanolic extracts, while flavonoids, saponins, and quinones were detected in the bark extract but not in the leaf extract. Oxalate was detected in the ethanolic and methanolic leaf extracts (EE and ME), but it was absent in the bark extracts and in the DW extracts of both plant parts. In the distilled water extracts, glycosides, phenols, saponins, and oxalate were not detected in the leaf extract, whereas these constituents were detected in the bark extract, except for oxalate. The presence of flavonoids and phenolic compounds, particularly in ethanolic extracts, is consistent with previous studies reporting their abundance in *C. macleodii* and their potential contribution to antioxidant activity.

**Table 5:** Total phenolic content (TPC) and total flavonoid content (TFC) of EE.

Plant parts	Extracts	Total phenolic content (mg CE/g)	Total flavonoid content (mg QE/g)
Bark	Aqueous	3.84	5.01
	Ethanolic	11.72	12.19
	Methanolic	8.12	9.81
Leaves	Aqueous	1.59	3.21
	Ethanolic	4.30	8.41
	Methanolic	3.92	6.22

### 3.4 Assessment of total phenolic and flavonoid content

**Table 5** presents the total phenolic content (TPC) and total flavonoid content (TFC) of *C. macleodii* leaf and bark extracts obtained using ethanol, methanol, and distilled water. A linear catechol calibration curve (R² = 0.967) was used to determine the TPC of the different extracts. The highest TPC was recorded in the ethanolic bark extract (EE) (11.72 mg CE/g), followed by the methanolic bark extract (ME) (8.12 mg CE/g). The lowest TPC was observed in the aqueous leaf extract (1.59 mg CE/g), followed by the aqueous bark extract. In contrast, the TFC values of the different plant material extracts (leaf and bark) ranged from 3.21 mg QE/g of dried sample in the aqueous leaf extract to 12.19 mg QE/g of dried sample in the methanolic bark extract, representing the lowest and highest values, respectively (**Table 5**).

## 4 Discussion

The effective categorization, sustainable utilization, and development of appropriate conservation strategies for endangered plant species, such as *C. macleodii*, require a comprehensive understanding of their taxonomic identity and phytochemical profile (Deb et al., 2018). *C. macleodii* is a medium-sized deciduous tree characterized by light brown bark that exfoliates into papery flakes and simple, alternate, ovate leaves measuring 8–16 cm in length. The leaves possess a cordate base and a greyish tomentose underside, representing distinctive morphological traits that differentiate this species from other *Cordia* taxa. In the genus *Cordia*, inflorescences are cymose and frequently arranged as open panicles, while several species produce white to creamy, pentamerous flowers with a sympetalous corolla. Floral merosity is generally five, although occasional four- or six-parted flowers have been documented, suggesting floral plasticity rather than confirmed adaptive evolution (Nowicke and Ridgway, 1973; Pätzold et al., 2022; Pandey et al., 2026b).

The species exhibits considerable taxonomic complexity due to overlapping morphological characteristics, interspecific variation, and the occurrence of dioecious floral structures. Since dioecy is relatively uncommon within *Cordia*, its presence in *C. macleodii* may reflect genetic regulation of sex expression as well as specialization associated with pollination mechanisms. Female flowers possess a functional four-celled ovary with a bifid stigma and a prominent style, whereas male flowers generally exhibit stamens with basifixed, sagittate anthers and a non-functional ovary. The genus is characterized by drupaceous, ovoid fruits containing a single exalbuminous seed; however, in *C. macleodii*, the apical hook of the fruit is considered an important diagnostic trait. Flowering begins during the first week of March, and the simultaneous occurrence of flowering and fruiting in some individuals indicates a possible overlap between reproductive phases. The reported anthesis period (7:30 am–10:30 am) suggests a specific temporal window for pollen release and stigma receptivity, with butterflies potentially contributing to pollination.

In addition, all plant organs contain naturally occurring phytochemicals, with biologically active compounds present in bark, leaves, stems, roots, flowers, fruits, seeds, and other tissues (Tiwari et al., 2011). Due to their biological importance, these phytochemicals, commonly regarded as secondary metabolites, have attracted considerable research interest. However, both the qualitative and quantitative composition of these compounds may vary considerably among different plant organs. *C. macleodii* is traditionally recognized for its therapeutic potential against various health disorders. Therefore, to better understand its medicinal value and contribute to conservation efforts, the present phytochemical investigation was conducted to characterize the phytochemical composition of leaf and bark extracts obtained using different solvents, including ethanol (EE), methanol (ME), and distilled water (DW).

The results demonstrated that the presence and concentration of phytochemicals varied significantly depending on both the plant organ analyzed and the extraction solvent employed. Irrespective of the plant part or solvent system, important secondary metabolites, including alkaloids, tannins, coumarins, and phenolic compounds, were consistently detected in all extracts through qualitative phytochemical screening (**Table 5**). The antibacterial, antioxidant, and therapeutic properties of these compounds have been widely recognized (Raghuvanshi et al., 2022). The bark represented a richer source of bioactive compounds, as evidenced by the broader phytochemical profile observed in ethanolic and methanolic bark extracts compared with leaf extracts. The detection of terpenoids and sterols exclusively in aqueous extracts, while absent from both alcoholic extracts, suggests that these compounds in *C. macleodii* may exhibit higher stability in polar aqueous environments or greater water solubility.

Furthermore, glycosides, phlobatannins, and quinones were detected only in bark extracts, indicating tissue-specific accumulation that may be associated with the protective and storage functions of bark tissues. Conversely, oxalates were identified exclusively in alcoholic leaf extracts and were absent from bark and aqueous extracts, reflecting differential compartmentalization and selective solubility patterns within plant tissues. Similarly, the predominance of bioactive compounds in bark tissues was further supported by quantitative assessments of total phenolic content (TPC) and total flavonoid content (TFC) (**Table 3**). The ethanolic bark extract exhibited a considerable phenolic concentration of 11.72 mg CE/g, followed by the methanolic bark extract with 8.12 mg CE/g.

Previous phytochemical investigations have reported higher concentrations in alcoholic leaf extracts, with phenolic content reaching up to 47.89 mg GAE/g and flavonoid content of 52.43 ± 3.21 mg QE/g. In comparison, bark extracts exhibited phenolic concentrations of 84.23 mg GAE/g and flavonoid levels of 18.56 ± 0.95 mg QE/g (Ahirwar et al., 2026). Conversely, Chaubey et al. (2015) reported considerably lower quantities of these phytochemicals in *C. macleodii* leaves and bark, with phenolic contents ranging from 0.65 to 1.65 mg GAE/g and flavonoid contents ranging from 0.985 to 1.89 mg QE/g in leaves and bark, respectively.

Ghasemzadeh and Ghasemzadeh (2011) reported that phenolic compounds, including simple phenolics, phenolic acids, anthocyanins, and flavonoids, exhibit strong antioxidant activity mainly due to their reducing properties, which enable them to act as singlet oxygen quenchers, hydrogen donors, and reducing agents. The present findings support previous studies by demonstrating considerable levels of phenolic and flavonoid compounds in *C. macleodii* extracts. Kapadiya et al. (2016) emphasized that phenolic compounds have received substantial scientific attention due to their diverse physiological activities, including anti-inflammatory, anti-mutagenic, and anti-carcinogenic effects, as well as their capacity for free radical scavenging.

The biological activities and traditional medicinal applications of *C. macleodii* leaf and bark extracts may therefore be attributed to the diverse phytoconstituents identified in these tissues. Collectively, these findings not only support the traditional ethnomedicinal relevance of *C. macleodii* bark but also provide scientific evidence for its pharmacological potential. The integration of taxonomic authentication with comprehensive phytochemical characterization presented in this study establishes a reliable foundation for the conservation, sustainable propagation, and targeted pharmacological investigation of *C. macleodii*, highlighting its potential as a valuable resource for future herbal and pharmaceutical applications.

## 5 Conclusions

This study represents an integrated report on the taxonomy and phytochemistry of *C. macleodii*, an endangered and ecologically valuable tree species from Central India. The results obtained in the present study indicate that the medicinal properties of *C. macleodii* may be associated with the diverse phytoconstituents present in its leaves and bark. The observations further confirmed that the phenolic compounds present in *C. macleodii* leaves and bark may play an important role in various biological activities. The plant represents a significant source of secondary metabolites, further supporting the scientific recognition of *C. macleodii* as a medicinally valuable species. Furthermore, the findings provide opportunities for future pharmacological investigations aimed at exploring the bioactive potential of this endangered plant. The distinct taxonomic characteristics identified in this study represent reliable tools for accurate species identification. Among the previously established taxonomic traits of Dahiman, the presence of dioecious floral structures represents a newly identified feature of this species. These findings provide a scientific basis for the conservation, propagation, and potential pharmacological exploration of *C. macleodii*.

## Data Availability

The original contributions presented in the study are included in the article/Supplementary Material. Further inquiries can be directed to the corresponding authors.
